# Supplementary material for: Later but Not Weaker: Neural Categorization of Native Vowels of Children at Familial Risk of Dyslexia
Source: Brain Sci. 2022 Mar 21;12(3):412. doi: 10.3390/brainsci12030412 (PMC8946763; doi:10.3390/brainsci12030412)
Supplement: Supplementary file 1 [file brainsci-12-00412-s001.zip › brainsci-1617563-supplementary.pdf]

Supplementary materials

Table S1. Time windows (ms after stimulus onset) where the standard and the deviant ERPs differed significantly for the multiple-speaker (MS) and single-speaker (SS) conditions. (+) indicates that the deviant ERP was more positive than the standard ERP, and (-) indicates that the deviant ERP was more negative than the standard ERP, and -- indicates lack of significant difference.

		F3	Fz	F4
TD	MS	296–500 (+)<br>576–596 (+)	--	264–288 (+)
	SS	336–392 (+)	572–596 (-)	--
FR	MS	204–584 (+)	204–252(+)<br>328–360(+)<br>376–404(+)<br>440–476(+)<br>492–504(+)	296–388 (+)
	SS	324–364(+)	--	--

Table S2. Mean (SD) amplitude (μv) of the standard ERP (STD), the deviant ERP (DEV), and the p-MMR in the multiple-speaker (MS) and single-speaker (SS) condition at corresponding p-MMR peak latencies, separated for the typically developing children (TD) and children at familial risk of dyslexia (FR).

			F3	Fz	F4
TD	MS	STD	0.87 (1.84)	0.63 (1.74)	2.66 (2.73)
		DEV	2.66 (2.73)	2.09 (2.32)	1.46 (2.70)
		p-MMR	1.78 (2.61)	1.46 (2.61)	0.58 (3.07)
	SS	STD	0.51 (2.36)	−0.07 (1.80)	−0.05 (2.06)

		DEV	2.09 (3.03)	0.89 (3.69)	0.85 (3.62)
		p-MMR	1.58 (3.21)	0.95 (3.74)	0.89 (4.16)
FR	MS	STD	0.70 (2.22)	0.19 (2.62)	0.47 (2.60)
		DEV	3.71 (3.59)	2.10 (4.01)	1.98 (3.12)
		p-MMR	3.01 (3.13)	1.91 (3.34)	1.51 (3.24)
	SS	STD	0.34 (2.10)	−0.15 (2.25)	0.15 (2.16)
		DEV	1.25 (2.64)	0.89 (2.83)	1.05 (3.42)
		p-MMR	0.91 (2.83)	1.04 (3.21)	0.90 (3.75)
